# On the Radical Diversity of the Vaccine and Variolous Diseases

**Published:** 1806-03

**Authors:** James John Namlat

**Affiliations:** Bromley, Kent


### On the Radical Diversity of the Vaccine and Variolous Diseases; *by* James John Namlat, *of Bromley, Kent.*

When Dr. Kinglake's propensity to assimilate the most opposite diseases is considered; when we recollect he maintains the identity of gout, white swelling, lumbago, sciatica, rheumatism, and sprain; (vide his Dissertation) when, not contented with these large strides into the regions of medical novelty, he has advanced a step further, and declared his assent to that wonderful proposition, which makes apoplexy to commence in a painful swelling and inflammation of the great toe, (vide his Observations on Mr. O'Neal's case,) we must cease to admire, that he should make so great a discovery as that which he announces in the last number of your Journal, *the radical identity* of the vaccine and variolous diseases. He is, however, rather unfortunate in the means by which he would persuade us of the radical *identity*; indeed, if we give to experiments and arguments their due weight, he could not have adduced stronger reasons to prove their radical *diversity*. No objection can be offered to the experiments, which he made to ascertain his proposition; they were conducted with great fairness, and sufficiently evince the impossibility of giving the variolous disease to the cow. The vaccine then, to which the cow is subject, is radically different from the variolous, which has never been observed in that animal, and which cannot be communicated to it. But it is supposed that the vaccine-pock is nothing more than a *mitigated form* of the variolous, in consequence of the blander fluids, or more moderate activity of the vaccine vessels, which produce it. This supposition is immediately refuted by transferring the disease to the human subject; for in that case the mitigated character of the vaccine should disappear, and the variolous be assumed, in consequence of the more acrimonious fluids or stronger activity of the human vessels, which are now actuated by that virus. But this is contrary to daily and hourly experience, which demonstrates that the vaccine disease, after reiterated inoculations into the human animal, still preserves its mild form, and peculiar characteristics, the reverse of what the variolous exhibits in so many respects, that no body but the identifier of gout and apoplexy, could possibly regard them as *kindred*, much less as the *same* diseases.

That the vaccine is a security against the variolous, no more proves their identity, than its being a security against the distemper in dogs, which has been asserted, identifies it with the latter malady.

It cannot be necessary to occupy your pages with a description of the numerous particulars in the two diseases, which oppose themselves to Dr. Kinglake's conclusion; such as the different size of the respective pustules arising from the *vaccine* virus and the *variolous*; the uninfectious nature of the former, the contagiousness of the latter; the single pock of the one, the dreadful plurality of the other; the perfect safety of the first at all ages and in every variety of constitution, and the too often experienced fatality of the second, under apparently the most favourable circumstances; but they are such as, I think, cannot fail to convince every one, who does not give way to the reveries of visionary physiology, of the *radical diversity* of these rival maladies.

*January* 16, 1806.

